# Clinical features and mutational frequency of renal cell carcinoma from patients undergoing kidney transplant evaluation

**DOI:** 10.3389/fonc.2025.1526545

**Published:** 2025-05-29

**Authors:** Corbin J. Eule, Junxiao Hu, Kurtis D. Davies, Alkesh Jani, Thomas Pshak, Elaine T. Lam

**Affiliations:** ^1^ Division of Medical Oncology, University of Colorado Anschutz Medical Campus, Aurora, CO, United States; ^2^ Biostatistics Core, University of Colorado Cancer Center, Aurora, CO, United States; ^3^ Department of Pathology, University of Colorado Anschutz Medical Campus, Aurora, CO, United States; ^4^ Division of Nephrology, University of Colorado Anschutz Medical Campus, Aurora, CO, United States; ^5^ Rocky Mountain Regional Veterans Administration Medical Center, Aurora, CO, United States; ^6^ Division of Urology, University of Colorado Anschutz Medical Campus, Aurora, CO, United States

**Keywords:** renal cell carcinoma, chronic kidney disease, end stage renal disease, kidney transplant, genomics

## Abstract

**Background:**

Patients with chronic kidney disease (CKD) or post-kidney transplant have an elevated risk of renal cell carcinoma (RCC). Despite increased understanding of genomic alterations in clear cell and papillary RCC, the most common subtypes, little is known about the effects of renal dysfunction on RCC pathogenesis.

**Materials and methods:**

This retrospective study analyzed electronic medical records and pathology data from adult patients with renal cell carcinoma (RCC) who were evaluated for or received a kidney transplant at a single institution between 1995 and 2021. Molecular sequencing of RCC tissue samples was conducted to compare mutation rates in patients with renal dysfunction compared with those reported by The Cancer Genome Atlas (TCGA).

**Results:**

This study identified 21 patients with RCC undergoing kidney transplant evaluation, mostly males with an increased occurrence of papillary RCC (38.1%) and early-stage disease (85.7%). Among clear cell RCC tumors, *SDHA* mutations were significantly more common compared to the TCGA cohort. For papillary RCC, genes such as *BRD4* and those involved in DNA damage repair demonstrated increased mutation rates in patients with renal dysfunction

**Conclusions:**

This study identifies key mutations in RCC among patients with CKD and post-kidney transplant, highlighting a complex relationship between renal dysfunction, inflammation, and tumorigenesis. Despite its limited sample size, the findings underscore the need for further research to understand the molecular drivers of RCC in high-risk populations, which could lead to more personalized treatment strategies.

## Introduction

In 2023, 27,332 kidney transplants were performed in the United States, constituting 81% of solid organ transplants in that year (1). Following solid organ transplantation, the risk of malignancy increases despite an improvement in overall survival (2–4). In particular, renal cell carcinoma (RCC) was found to have a standard incidence ratio (SIR) of 4.7 as compared to the general population (p <0.001) in a cohort of over 175,000 solid organ transplant patients between 1987-2008. This is greater than the SIR of 2.10 (2.06-2.14 95% CI) of all malignancies in patients who have undergone a solid organ transplant (4). In a general population, the most prevalent histopathologic subtype of RCC is clear cell RCC (approximately 80 percent) (ccRCC) followed by papillary (15 percent) and chromophobe (5–10 percent) RCC (5–7). For kidney transplant recipients, the relative risk for RCC is highest for papillary RCC (SIR 13.3 vs. 3.98 for ccRCC) (8). While the increased frequency of abdominal imaging for patient undergoing a kidney transplant evaluation may facilitate RCC detection, this alone does not account for the increased RCC risk in the transplant patient population (9).

While the increased incidence of RCC following kidney transplant is an increasingly recognized phenomenon, the contribution of specific risk factors is not well elucidated. Chronic kidney disease (CKD) and acquired cystic kidney disease are associated with an increased risk of RCC (10, 11). End-stage renal disease (ESRD), kidney transplant, and impaired kidney graft function have also been shown to increase RCC risk (12, 13). However, RCC arising in patients with ESRD may have a more clinically indolent behavior with lower rates of metastasis and longer cancer-specific survival (14). Nonetheless, the genomic features of RCC arising in the context of CKD and ESRD and whether they differ from RCC in patients from the general population is not clearly understood (11, 15).

The Cancer Genome Atlas (TCGA) Research Network identified significantly mutated genes (SMGs) across over 500 ccRCC and papillary tumor specimens (16, 17). In ccRCC, the most common of these mutations occurred in the von Hippel Lindau (*VHL*) tumor suppressor gene implicated in cellular oxygen sensing and in the protein polybromo 1 (*PBRM1*) gene controlling the maintenance of chromatin (16). Clear cell RCC occurring in patients with ESRD may have lower rates of chromosome 3p loss, the location of *VHL* (18, 19). Studies of molecular alterations in RCC and ESRD have primarily been limited to chromosomal analysis (20). To our knowledge, no prior study has investigated the incidence of RCC gene mutations in the pre- and post-kidney transplant settings.

RCC is a significant contributor to the morbidity and mortality of patients with renal disease and following kidney transplant. Our study aims to characterize the clinical and genomic features of RCC as they relate to patients in the pre-kidney and post-kidney transplant settings at a kidney transplant center and identify potential differences in sporadic cases of RCC from TCGA cohort.

## Materials and methods

### Study design and patients

This is a retrospective study with collection of secondary use clinical and pathologic data. The electronic medical record (EMR) was queried for adult patients with a confirmed pathologic diagnosis of RCC who have (1) undergone formal evaluation for a kidney transplant and/or (2) received a kidney transplant at a single institution between 1995-2021. Patients who received multi-organ transplants were excluded from this study.

Demographic, clinical, pathologic, molecular, and outcomes data were extracted from the EMR. Standardized data collection templates were used to minimize inter-observer variation. Available RCC tissue specimens from the identified pre-kidney and post-kidney transplant were collected for molecular sequencing. The primary endpoint of this study was mutation rates of clear cell and papillary RCC in patients with CKD relative to mutation rates reported by TCGA (accessed September 27, 2023). This study was submitted to the Colorado Multiple Institutional Review Board (21-4627) and determined to be exempt from review due to the use of secondary data.

### Molecular analysis

Archived tumor material was retrieved and then reviewed by a board-certified pathologist to determine adequacy for molecular testing. Tumor enrichment from samples was achieved by a pathologist-guided microdissection or macrodissection procedure prior to nucleic acid extraction. Mutational analysis of extracted total nucleic acid (TNA) was performed using a clinically validated next-generation sequencing (NGS)-based assay that assesses mutational status across all coding regions of 498 genes. Target enrichment for this assay was achieved through a hybrid-capture approach and sequencing was performed on the Illumina NextSeq platform. Raw NGS data was processed using a custom bioinformatic analysis pipeline and all variant calls were manually inspected by an expert (author KDD). Mutations in TCGA were identified using whole exome sequencing, copy-number analysis, messenger RNA and microRNA sequencing, DNA-methylation analysis, and proteomic analysis (16, 17).

### Statistical methods

Summary statistics were reported for clinical and demographic characteristics. Continuous variables were presented with median and IQR. The categorical variables were presented with frequency and percentages. For each mutation and cohort, the frequencies and the percentages were calculated. Fisher’s exact test were conducted to compare the mutation rates between the two groups for clear cell tumors (all clear cell tumors biopsies were collected independently from patients). The permutation test based on log likelihood ratio were conducted compare the mutation rates between the two groups for papillary tumors (2 tumor biopsies were collected from one patient with bilateral papillary RCC at the same time, and the remaining biopsies were collected independently from patients), to address the clustered biopsies for the one patient with bilateral papillary RCC.

Statistical significance level was set to 0.05. All statistical analysis were conducted using R version 4.1.0, R Core Team (21).

## Results

A total of 21 patients who underwent kidney transplant evaluation and had a diagnosis of RCC were identified (**Table 1**). The patients in the cohort include those with CKD, ESRD on dialysis, or post-kidney treansplant at the time of RCC diagnosis. The majority of patients were male (71.4%) and non-Hispanic white (61.9%). An increased proportion of papillary RCC histology was present (38.1%). RCC was most frequently diagnosed as early disease (stage I, 85.7%). Most RCC tumors were diagnosed in dialysis patients (76.2%) with a median dialysis duration of 64.7 months. The 24 RCC specimens, 12 ccRCC and 12 papillary RCC were collected from 21 patients. Two patients had metachronous clear cell and papillary RCCs, and one patient had bilateral papillary RCCs at the time of resection. Two patients had ccRCC of the allograft kidney and the remainder of RCCs were isolated from the native kidney. Both patients with ccRCC of the allograft kidney did not have BK virus detected on PCR. The majority (18 patients, 85.7%) were diagnosed with stage I tumors.

**Table 1:** Baseline clinical and demographic characteristics of patients evaluated for kidney transplant at time of RCC diagnosis.

Total Patients		n = 21 (%)
Age at first RCC diagnosis (years)	Years (median, IQR)	54.4 (38.5 to 64.0)
Sex	Female	6 (28.6)
	Male	15 (71.4)
Race and Ethnicity	Non-Hispanic White	13 (61.9)
	Non-Hispanic Black	4 (19.0)
	Hispanic	3 (14.3)
	Asian	1 (4.8)
RCC Histology per Individual Patient	Clear Cell RCC	10 (47.6)
	Papillary RCC	8 (38.1)
	Clear Cell RCC and Papillary RCC	2 (9.5)
	Bilateral Papillary RCC	1 (4.8)
Anatomic Stage at first RCC Diangosis	I	18 (85.7)
	II	0
	III	1 (4.8)
	IV	1 (4.8)
Kidney Dysfunction at first RCC Diagnosis	CKD Stage 3b	1 (4.8)
	CKD Stage 4	1 (4.8)
	CKD Stage 5	1 (4.8)
	ESRD on dialysis	10 (47.6)
	Post-kidney transplant	8 (38.1)
Dialysis Prior to first RCC Diangosis	Yes	16 (76.2)
	No	5 (23.8)
Time on Dialysis (months)	Median (IQR)	64.7 (36.8 to 92.3)

RCC, renal cell carcinoma; CKD, chronic kidney disease; ESRD, end-stage renal disease; IQR, interquartile range.

Among the 12 ccRCC tumors collected from patients with renal dysfunction, there was no significant difference in the SMGs identified by TCGA (**Table 2**). The gene *SDHA* was mutated in 3 ccRCC tumors (25.0%) in patients with renal dysfunction but no patients from TCGA cohort, which was statistically significant (p <0.001). Five additional genes were mutated at greater frequency in patients with RCC and renal dysfunction compared to TCGA cohort.

**Table 2:** Mutation rates in clear cell RCC of the most frequently mutated genes in TCGA and renal dysfunction cohorts.

Cohort	Mutated Gene	Renal Dysfunction	TCGA	P-value
	Total *n* (%)	12 (3.1)	370 (96.6)	
Most Frequent Mutations in TCGA Cohort	*VHL*	8 (66.7)	170 (45.9)	0.239
	*PBRM1*	3 (25.0)	152 (41.1)	0.374
	*SETD2*	0 (0.0)	45 (12.2)	0.374
	*BAP1*	0 (0.0)	41 (11.1)	0.626
	*MTOR*	0 (0.0)	28 (7.6)	1.000
	*KDM5C*	0 (0.0)	20 (5.4)	1.000
	*KMT2C*	1 (8.3)	18 (4.9)	0.463
	*ATM*	0 (0.0)	16 (4.3)	1.000
Most Frequent Mutations in Renal Dysfunction Cohort	*SDHA*	3 (25.0)	0 (0.0)	<0.001
	*IRS2*	3 (25.0)	1 (0.3)	<0.001
	*SPTA1*	3 (25.0)	8 (2.2)	0.003
	*BUB1B*	2 (16.7)	4 (1.1)	0.013
	*FAT1*	2 (16.7)	11 (3.0)	0.058
	*NCOA2*	2 (16.7)	3 (0.8)	0.009

Among patients with renal dysfunction and papillary RCC (12 tumors collected), again there was no significant difference from the most commonly mutated genes identified by TCGA (**Table 3**). In the renal dysfunction cohort, multiple genes had increased mutation rates relative to TCGA, with mutations in *BRD4* and *CHEK2* being the most frequent.

**Table 3:** Mutation rates in papillary RCC of the most frequently mutated genes in TCGA and renal dysfunction cohorts.

Cohort	Mutated Gene	Renal Dysfunction	TCGA	P-value
	Total *n* (%)	12 (4.1)	278 (95.9)	
Most Frequent Mutations in TCGA Cohort	*MET*	1 (8.3)	20 (7.2)	1.000
	*KMT2C*	0 (0.0)	19 (6.8)	0.472
	*KMT2D*	1 (8.3)	17 (6.1)	1.000
	*FAT1*	1 (8.3)	16 (5.8)	1.000
	*SETD2*	0 (0.0)	15 (5.4)	0.644
	*BAP1*	0 (0.0)	13 (4.7)	0.665
	*ARID1A*	0 (0.0)	13 (4.7)	0.665
Most Frequent Mutations in Renal Dysfunction Cohort	*BRD4*	3 (25.0)	4 (1.4)	0.002
	*CHEK2*	3 (25.0)	2 (0.7)	0.001
	*LRP1B*	2 (16.7)	8 (2.9)	0.059
	*BRCA2*	2 (16.7)	6 (2.2)	0.038
	*CSF3R*	2 (16.7)	2 (0.7)	0.009
	*FAT3*	2 (16.7)	3 (1.1)	0.015
	*HNF1A*	2 (16.7)	3 (1.1)	0.015
	*KMT2A*	2 (16.7)	6 (2.2)	0.038
	*MAGI2*	2 (16.7)	5 (1.8)	0.029
	*MST1R*	2 (16.7)	2 (0.7)	0.009
	*PREX2*	2 (16.7)	2 (0.7)	0.009
	*PRKDC*	2 (16.7)	8 (2.9)	0.059
	*RARA*	2 (16.7)	0 (0.0)	0.002
	*RPTOR*	2 (16.7)	1 (0.4)	0.005
	*SAMD9*	2 (16.7)	4 (1.4)	0.022
	*TRAF7*	2 (16.7)	0 (0.0)	0.002
	*U2AF1*	2 (16.7)	0 (0.0)	0.002
	*XPC*	2 (16.7)	0 (0.0)	0.002
	*ZNF750*	2 (16.7)	0 (0.0)	0.002

## Discussion

This study investigated the molecular underpinnings of RCC in patients with CKD and post-kidney transplant, focusing on the genetic mutations that may influence tumorigenesis and treatment outcomes. Among 21 patients undergoing kidney transplant evaluation with RCC, the majority were male, non-Hispanic white, diagnosed with early-stage RCC prior to kidney transplantation and following long-term dialysis. TCGA cohort similarly showed a male predominance, although with a larger proportion of advanced RCC disease (22). The median time on hemodialysis was 64.7 months (IQR 36.8, 92.3). Notably, no *BAP1* mutations were detected in the renal dysfunction cohort. *BAP1* loss correlates with more clinically aggressive sporadic ccRCC given its association with high tumor grade and worse survival outcomes for patients (23). While not statistically significant, this finding corresponds with our previous work demonstrating lower rates of *BAP1* mutations in RCC from patients with advanced CKD relative to patients without significant renal dysfunction (24).

SDH-deficient RCC is very rare, estimated to make up less than 0.2% of all RCCs (25). SDH-deficient RCC is most often due to a mutation in *SHDB*, while *SDHA-*deficient RCC is exquisitely rare (26). However, in ccRCC specimens from the renal dysfunction cohort, *SDHA* was mutated in 25.0% of patients. *SDH*, which encodes the succinate dehydrogenase complex, is a tumor suppressor gene involved in the mitochondrial respiratory chain and conversion of succinate to fumarate in the citric acid cycle (25, 26). Although SDH-deficient RCC in uncommon, functional *SDH* loss may play a broader role in RCC pathogenesis and was found to be a common adverse feature in approximately 80% of ccRCC cases (27). Succinate accumulation during renal ischemia is oxidized during reperfusion, yielding excessive reactive oxygen species and kidney damage (28). Our findings suggest that *SDHA* dysfunction may contribute to both CKD and ccRCC tumorigenesis through a similar underlying process.

*BRD4* was one of the most frequently mutated genes in papillary RCC from patients with renal dysfunction. *BRD4*, which encodes bromodomain-containing protein 4, is a member of the bromodomain and extraterminal (BET) protein family and is essential to chromatin structure formation, transcription elongation, and epigenetic regulation (29). BRD4 binding leads to sustained activation of the transcription factor nuclear factor-κB (NF-κB), leading to aberrant cell proliferation (30). BRD4 and other BET proteins have been implicated in the upregulation of inflammatory genes in CKD, and BET inhibitors have been studies in preclinical setting for treatment of kidney injury and prevention of renal fibrosis (31). The impact of *BRD4* in tumorigenesis and development of renal disease may illustrate an overlapping role in RCC formation in CKD.

In papillary RCC isolated from patients with renal dysfunctions, higher rates of *CHEK2*, *BRCA2*, and *XPC* mutations, all genes involved in DNA damage repair (DDR), were observed. Additionally, 3 other papillary RCC tumors were found to have a mutation in the DDR genes *BRCA1*, *RAD51B*, or *MLH3*. One papillary RCC had coexisting *BRCA2* and *NDN* mutations. DDR response in renal tubular cells in response to acute injury has been shown to determine whether cellular recovery or irreversible damage occurs (32). In particular, loss of DDR is associated with irreversible kidney injury in cisplatin-exposed kidney organoid model (32). Considering the known association between CKD and papillary RCC, this raises the questions whether loss of DDR may be a mutually reinforcing process which facilitates progressive renal dysfunction and RCC tumorigenesis (8). The impact of DDR alterations on RCC is the premise of an ongoing clinical trial examining whether the poly ADP ribose polymerase inhibitor (PARPi) olarapib has antitumor activity in RCC harboring *BAP1* or DDR mutations (NCT03786796).

Interestingly, in the renal dysfunction cohort, *TRAF7* and *RPTOR* were mutations that were detected in the patient with bilateral papillary RCC diagnosed post-kidney transplant. *TRAF7*, TNF receptor associate factor 7, encodes an E3 ubiquitin ligase that potentiates MEKK3-mediated apoptosis (33). *TRAF7* has previously been identified in NF2-independent meningiomas, malignant mesothelioma, and in small numbers of clear cell, papillary, and chromophobe RCCs (34, 35). *TRAF7* has a role in the regulation of innate and adaptive immune responses, but it is indeterminate whether the post-transplant tumor environment or immunosuppression may enrich for this mutation (34). *RPTOR*, regulatory associated protein of mechanistic target of rapamycin (mTOR) complex 1, is a gene in the PI3K/AKT/mTOR pathway which is up regulated following loss of *VHL* (36). The mTOR inhibitor everolimus is an efficacious subsequent line option for the treatment of advanced RCC (37). Additionally, the mTOR inhibitors sirolimus and tacrolimus are commonly used immunosuppressive medication to prevent post-kidney transplant rejection (38). The degree to which immunosuppression impacts RCC risk, including potential molecular alterations, remains unclear (39). The most commonly used immunosuppressive regimen in this cohort was tacrolimus, mycophenolate, and prednisone.

Due to the small sample size, these results should be interpreted primarily as hypothesis generating, but collectively illustrate distinct molecular underpinnings for clear cell and papillary RCC pathogenesis in patients with renal dysfunction and following a kidney transplant. Mutational results for the renal dysfunction cohort were limited to those in the 498-gene panel, which by nature is less comphrehensive than bioinformatics studies utilizing a combination of whole genome, whole exome, transcriptome sequencing, or other molecular analysis. Germline genetic testing was not conducted for the patients in this study, limiting the ability to correlate it with the observed somatic mutations; however, this impact is expected to be minimal. At last follow-up, no patients had died as a result of their RCC, limiting the potential to detect a correlation between genes with high mutation rates and cancer-specific survival.

This study highlights distinct molecular characteristics of RCC in patients with CKD and post-kidney transplant, identifying key mutations such as *SDHA*, *BRD4*, and DDR-related genes *CHEK2* and *BRCA2*. These mutations suggest a complex interplay between renal dysfunction, tumorigenesis, inflammation, and immune modulation. The findings illustrate that CKD and RCC may share overlapping mechanisms, potentially contributing to both renal injury and cancer development. Although the study is limited by its small sample size, it underscores the importance of continued research to better understand the molecular drivers of RCC in these high-risk populations. Future studies could ultimately inform more personalized and effective treatment strategies and clarify the influence of specific mutations on RCC-specific survival outcomes.

## Data Availability

The data supporting the findings of this study are available from the corresponding author upon reasonable request and completion of a data sharing agreement.
